# The Role of Single Positive Cultures in Presumed Aseptic Total Hip and Knee Revision Surgery—A Systematic Review of the Literature

**DOI:** 10.3390/diagnostics13091655

**Published:** 2023-05-08

**Authors:** Jan Schwarze, Burkhard Moellenbeck, Georg Gosheger, Jan Puetzler, Niklas Deventer, Tobias Kalisch, Kristian Nikolaus Schneider, Sebastian Klingebiel, Christoph Theil

**Affiliations:** Department of General Orthopedics and Tumor Orthopedics, University Hospital Muenster, Albert-Schweitzer-Campus 1, 48149 Muenster, Germany

**Keywords:** periprosthetic joint infection, revision arthroplasty, aseptic revision, PJI, total knee revision, total hip revision, arthroplasty, unsuspected positive cultures, unexpected positive cultures

## Abstract

(1) Background: Prior to revision hip (THA) or knee arthroplasty (TKA), periprosthetic low-grade infection (PJI) should be ruled out. Despite advances in preoperative diagnosis, unsuspected positive cultures (UPCs) may occur in initially planned aseptic revisions. Particularly, single UPCs pose a diagnostic and therapeutic dilemma, as their impact on outcome is unclear and recommendations are heterogeneous. This review investigates the frequency of single UPCs and their impact on implant survivorship. (2) Methods: In July 2022, a comprehensive literature search was performed using PubMed and Cochrane Library search. In total, 197 articles were screened. Seven retrospective studies with a total of 5821 cases were able to be included in this review. (3) Results: Based on the cases included, UPCs were found in 794/5821 cases (14%). In 530/794 cases (67%), the majority of the UPCs were single positive. The most commonly isolated pathogens were coagulase negative *Staphylococci* and *Cutibacterium acnes*. Five of seven studies reported no influence on revision- or infection-free survival following a single positive culture. In two studies, single UPCs following THA revision were correlated with subsequent re-revision for PJI. (4) Conclusions: Single UPCs of a non-virulent pathogen following presumed aseptic TKA revision may be interpreted as contaminants. A single UPC following THA revision may be a risk factor for subsequent PJI. The role of systemic antibiotic treatment remains unclear, but it should be considered if other risk factors for PJI are present.

## 1. Introduction

Revision surgery for total knee arthroplasty (TKA) and total hip arthroplasty (THA) is estimated to increase by 332% and 137%, respectively, from 2012 to 2030 [1]. Prior to planned THA or TKA revision, periprosthetic joint infection (PJI) should to be ruled out [2]. PJI following THA and TKA revision is a leading cause for repeat revision surgery and is associated with disastrous consequences, such as immobilization of the patient, amputations, and increased mortality of up to 21% after 5 years [3,4,5,6,7,8,9,10]. Therefore, PJI frequently results in prolonged hospitalization of the patient due to repeat revision surgery and antibiotic treatment, causing high costs for the health care system and the community [8,9].

PJI is usually diagnosed using validated criteria, such as criteria published by the Musculoskeletal Infection Society (MSIS), the International Consensus Meeting (ICM), the European Bone and Joint Infection Society (EBJIS), the Infectious Diseases Society of America (IDSA), and the American Academy of Orthopaedic Surgeons (AAOS) [2,11,12,13,14,15,16]. These frequently updated diagnostic algorithms consist of a combination of clinical signs of infection, inflammatory parameters in blood serum and synovial fluid, and microbiological findings in the preoperative joint aspirate and intraoperative samples, including histological investigation as well. Although numerous diagnostic algorithms for the diagnosis of a PJI of THA and TKA exist, the diagnostic accuracy varies, and there is no “gold standard” for the preoperative diagnosis of a possible “low-grade” PJI as an underlying cause of prosthetic failure.

The World Association against Infection in Orthopedics and Trauma (WAIOT) proposed a definition of PJI regarding clinical appearance [17]. While patients with acute or “high-grade” PJI often present with suspicious clinical signs for infection such as fever, local swelling, dermal erythema, and pain, and further diagnostics often provide elevated infection parameters in the blood serum, patients with chronic or “low-grade” PJI may lack clinical signs of infection, and serum infection parameters may be normal [6,18,19]. Therefore, the correct diagnosis in cases of chronic or “low-grade” infections of TKA and THA is challenging and a topic of high interest among orthopedic surgeons.

Furthermore, in patients with PJI, isolation of a causative pathogen in the joint aspirate can be difficult, as microbiological cultures from synovial fluid vary in their accuracy of the identification of the causative pathogen. The reported sensitivity of aspiration cultures ranges from f 12 to 94% [20]. In addition, the combination of preoperative microbiological and histopathological sampling has recently been associated with elevated sensitivity for the detection of PJI [21]. Nonetheless, even if the preoperative workup is not suspicious for PJI, the intraoperative collection of three to five tissue samples for a following microbiological investigation is recommended during THA and TKA revision [22,23]. Therefore, unsuspected positive cultures (UPCs) may occur in routinely collected tissue samples during THA and TKA revision preoperatively classified as aseptic [24].

UPCs may occur in up to 37% of planned aseptic THA and TKA revisions [24]. These UPCs have been of rising interest to orthopedic surgeons in the last decade because they have been associated with subsequent PJI while underlying patient-, procedure-, or implant-related risk factors are vastly unknown. In the current literature, male sex, obesity, chronic kidney disease, benign prostate hyperplasia, elevated serum C-reactive protein (CRP), and revision for adverse metal reaction and component loosening are discussed as possible risk factors for finding UPCs or subsequent revision for PJI [25,26,27,28,29,30,31,32].

Two or more UPCs of the same pathogen have been associated with decreased infection-free and revision-free survival following planned THA and TKA revision [24,33,34,35]. The most commonly reported pathogens isolated in patients with UPCs are low-virulent bacteria such as *coagulase-negative Staphylococci* and *Cutibacterium acnes* [24,33,36,37,38,39,40,41,42].

In contrast, the role of single UPCs remains a subject of debate regarding their impact on infection-free survival following aseptic THA and TKA revision. While the IDSA defines a single positive culture of a high-virulent pathogen (e.g., *Staphylococcus aureus*) as possible evidence for PJI, occurrence of a single positive culture of a low-virulent organism like *coagulase-negative Staphylococci* (*CoNS*) or *Cutibacterium acnes* is often considered to be a possible contamination of the specimen [12]. In contrast, there is growing evidence that single positive cultures may be associated with a higher risk of reinfection and repeat revision surgery [43,44].

In the current clinical practice, the MSIS and ICM guidelines define a single positive culture as a “minor criteria” for PJI, adding two points to a score classifying a joint as “infected” if it reaches six or more points [2,15]. In addition, the EBJIS criteria consider a single positive culture in an “infection likely” scenario if a second parameter (e.g., positive clinical feature or CRP) is positive [13].

The goal of this literature review is to systematically analyze the impact of single positive UPCs on infection-free survival following planned aseptic THA and TKA revision surgery.

## 2. Materials and Methods

**Figure 1:**
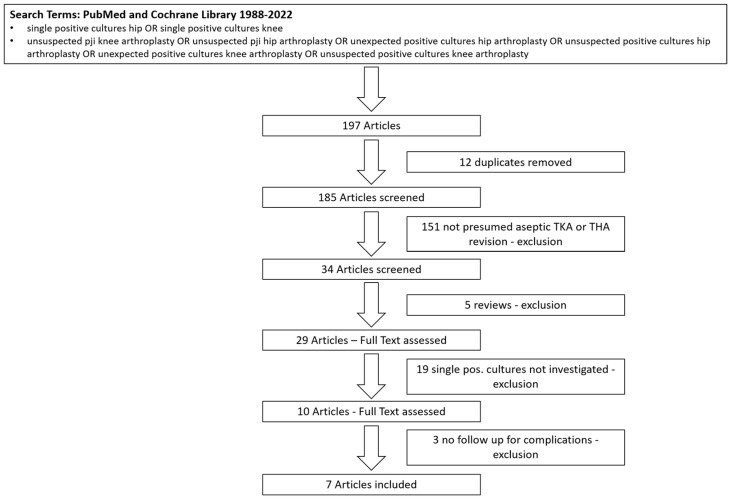
PRISMA Flow Diagram.

A comprehensive literature research of publications prior to 20 July 2022 was performed using the PubMed and Cochrane Library search. Search Terms were “single positive cultures hip OR single positive cultures knee”, and “unsuspected pji knee arthroplasty OR unsuspected pji hip arthroplasty OR unexpected positive cultures hip arthroplasty OR unsuspected positive cultures hip arthroplasty OR unexpected positive cultures knee arthroplasty OR unsuspected positive cultures knee arthroplasty”. The search was restricted to studies on humans published between 1988 and July 2022 for papers in English. The review algorithm was based on the updated Preferred Reporting Items for Systematic Review and Meta-Analyses 2020 (PRISMA) guideline [45], and search results are presented in a PRISMA flow diagram (Figure 1).

Titles and abstract were reviewed independently by two authors (Jan Schwarze and Christoph Theil). Following exclusion based on title and abstract, a full text was obtained and reviewed. The study quality of included research was determined using the Methodological Index for Non-Randomized Studies (MINORS) checklist [46] to display a quality score. The level of evidence was assessed using the Oxford Centre for Evidence-Based Medicine (OCEBM) levels of evidence (OCEBM Levels of Evidence Working Group, 2011).

Unsuspected positive cultures were defined as the isolation of a pathogen from intraoperative cultures taken during THA or TKA revision preoperatively classified as aseptic.

Studies were included if they reported UPCs in presumed aseptic THA or TKA revision. Studies in which single positive cultures were not investigated or declared as contamination and therefore classified as “sterile” were excluded. Only studies analyzing complications of any kind (such as re-revision) following single UPCs were included. Case reports or reviews were not included in this study.

We investigated the number of included cases, preoperative workup, type and amount of taken samples in revision surgery, amount of single UPCs, type of pathogens, therapeutic consequences following a single UPC, and follow-up for complications.

Statistical analysis was performed using Microsoft Excel (Microsoft Corporation, Redmond, WA, USA) and SPSS Statistics for Windows Version 25 (IBM Corporation, Armonk, NY, USA).

## 3. Results

In total, 197 articles were screened. Applying the inclusion criteria, seven studies were included in this review (Figure 1). All studies had a retrospective design and were classified as level 3 according to OCEBM (OCEBM Levels of Evidence Working Group, 2011). One registry-based study was included; of the remaining six studies, one was a multi-center study, and five studies investigated data of a single-center origin. Three studies investigated revision THA, two investigated TKA, and two studies included THA and TKA (Table 1).

The number of samples taken was reported in all seven studies. In most studies (six of seven), three to five intraoperative samples were taken during revision surgery (Table 2).

Five of the included studies reported an average follow-up ranging from 40 to 68 months (Table 2). The minimum follow-up for subsequent complications following a single UPC was 12–60 months (Table 2). Information on the preoperative diagnostic algorithm to rule out PJI before revision surgery was available in six of seven studies and mostly included clinical examination, plain radiographs, examination of serum infection parameters such as CRP, and, in most cases (five of seven), a selectively performed synovial fluid aspiration. A routinely performed aspiration of synovial fluid prior to revision surgery was performed in one study (Table 3).

Of all included 5821 presumed aseptic THA or TKA revisions, UPCs occurred in 794 cases (14%). A total of 67% (530/794) of UPCs were single positive (Table 4).

The most commonly isolated pathogens were low-virulent bacteria, predominantly CoNS and *Cutibacterium acnes* (*C. acnes*).

Of the included seven studies, five reported no statistically significant influence on the revision- or infection-free survival following a single positive culture [24,25,33,47,48]. Barrack et al. found that in 29 cases with a single UPC, no revision surgeries were performed [33]. In the cohort of Vargas-Revéron et al., patients with two or more UPCs had a higher re-revision rate after 5 years (23.1% vs. 3.2%). However, in their study, a single UPC was not associated with an increased risk for re-revision [47]. Neufeld et al. reported infection-free survival after two and five years for their entire cohort of 93.1% and 86.8%, respectively. In THA revisions, the rate of survivorship free from infection with the same organism cultured during presumed aseptic revision was 93.7% after five years [25]. For revision TKA, Neufeld et al. reported infection-free survival rates of 97.5% and 95.3% after two and five years, respectively. Again, the rate of survivorship free from infection with the same organism cultured during presumed aseptic revision was 98.7% after five years. They reported no statistically significant difference in the five-year infection-free survival in patients with a single UPC and ≥two positive UPCs [48]. In both their studies, cases with all sterile intraoperative cultures were not included to be used as a control group [25,48]. In contrast, Schwarze et al. included patients with all negative cultures as a control group. In their study, a single UPC had no impact on the revision-free survival rate after two and five years compared to the control group (66.2% vs. 76.7% and 59.8% vs. 70.6%, respectively, *p* = 0.13) after THA revision or TKA revision (78.4% vs. 76.6% and 59.9% vs. 63.5%, respectively, *p* = 0.85). In addition, the infection-free survival rate after two years following THA revision (90.9% vs. 95.4%, *p* = 0.17) and TKA revision (91.9% vs. 96.7%, *p* = 0.79) showed similar results for a single UPC compared to the sterile control group [24].

Information regarding antibiotic therapy following a single UPC was available in four of seven studies. In the cohort of Barrack et al. only 5 of 29 single UPC cases received antibiotic treatment for 4 to 6 weeks, and no patient underwent revision surgery [33]. In the study of Vargas-Reverón et al., 6 of 35 cases of single UPCs received antimicrobial therapy for 4 weeks [47]. In contrast, Hipfl et al. reported that none of the 41 single UPC cases received antibiotic treatment, as they were considered to be contaminations [35]. Neufeld et al. (2021 and 2022) did not specify in which cases (single or ≥two UPCs) antibiotic treatment was commenced [25,48]. Schwarze et al. reported that in 59 of 119 (50%) cases, antibiotic treatment was started following a single UPC. Furthermore, antibiotic therapy of a single UPC had no influence on the 2 years of infection-free survival in their cohort [24].

In the included registry-based study by Milandt et al. and the single center study by Hipfl et al., a single UPC following THA revision was correlated with increased risk for re-revision for PJI compared to culture-negative revisions and those with ≥two UPCs [35,44]. In detail, Hipfl et al. in their study on revision THA reported a seven-year survival rate of 97.5% in patients with negative cultures, 87.4% for a single UPC, and 94.4% for patients with ≥two UPCs [35]. In addition to these results, Milandt et al. reported an increased risk for re-revision due to PJI for single UPCs compared to the sterile control group, with a relative risk (RR) of 2.63 (95% CI 1.16–5.96), and when excluding “mixed growth revision”, the RR was 2.44 (95% CI, 1.02–5.84) for single UPCs in their cohort [44].

## 4. Discussion

Although a variety of established and validated diagnostic criteria for PJI exist, orthopedic surgeons may be confronted with UPCs in revision arthroplasty that was previously classified as aseptic. Previous studies suggested postoperative antibiotic treatment for PJI if two or more positive cultures of the same pathogen occurred.

In this systematic review, a single UPC in revision TKA had no influence on infection-free survival. In contrast, single positive UPCs following THA revision were correlated with subsequent PJI in two studies [35,44]. Interestingly, even compared to patients with ≥two UPCs, re-revision for PJI was more likely. Unfortunately, information on the causative pathogen in the subsequent re-revision for PJI was not available in these two studies, making further studies necessary to discriminate PJI persistence from possible recurrent PJI with a different pathogen. Neufeld et al. (2021 and 2022) also investigated infection-free survival, defining “PJI with the same microorganism” as the primary endpoint. However, they did not include aseptic cases as a control group and therefore limited their results to comparing single UPCs to ≥two UPCs [25,48].

Previous studies reported implant- and patient-related risk factors for PJI following revision arthroplasty, including adverse metal reaction, use of megaprosthesis, implant loosening, elevated serum CRP prior to revision surgery, obesity, and male sex [24,25,32,49]. Staats et al. reported a reduced implant survival rate in patients with a MSIS minor criteria for PJI (such as single UPCs) in combination with implant loosening compared to those without radiological signs of component loosening [31]. Since none of the included studies investigated the abovementioned combination of possible risk factors following PJI, a confounder bias for a single UPC as a risk factor for subsequent prosthetic failure due to PJI may be possible. In this context, further investigation of a combination of known patient- and implant-related risk factors for PJI and single UPCs may reveal new insights in terms of differentiation between contamination, colonization, and infection.

Sonication of THA and TKA components has been reported to improve the isolation of pathogens in revision arthroplasty, as it provides higher sensitivity compared to conventional tissue samples [50,51]. To further clarify the role of single positive UPCs, implant sonication in combination with tissue samples may offer additional information regarding the discrimination of possible contamination of the specimen and unsuspected low-grade PJI. In the included seven studies, only one (Hipfl et al. [35]) used implant sonication.

Furthermore, to discriminate contamination of the specimen from chronic low-grade PJI, additional histopathological investigation of the periprosthetic soft tissue as published by Krenn et al. may offer significant information in cases of single positive UPCs [52]. Alternatively, microscopic count of neutrophils per high-power field can be used as recommended in the MSIS and ICM criteria [2,15].

Despite the fact that this review was performed systematically, several limitations have to be mentioned. The results of this review may be limited by the small number of included studies. Although the inclusion criteria were very specific, the included studies were heterogeneous regarding their methods and interpretation of results. In particular, the number of taken samples, types of samples, and criteria for the use of antibiotic therapy following a single UPC were not consistent among the included studies. Furthermore, all included studies were of a retrospective design. Therefore, the results of this review should be interpreted with caution and not be extrapolated indiscriminately.

The results of this review highlight the need for further studies investigating single UPCs, especially in cases of THA revision since contradictory results are reported in the literature.

## 5. Conclusions

A single UPC of a non-virulent pathogen following presumed aseptic TKA revision may be interpreted as a contaminant in absence of further criteria for PJI. In contrast, a single UPC following THA revision previously classified as aseptic may be a risk factor for subsequent PJI. The included literature does not provide a general recommendation regarding antibiotic treatment of single UPCs following THA or TKA revision.

## Figures and Tables

**Table 1:** Included Articles.

Date	Author	Journal	Title	Type	Arthroplasty	OCEBM Level of Evidence	MINOR Score
2007 September	Barrack et al. [33]	Journal of Arthroplasty	The fate of the unexpected positive intraoperative cultures after revision total knee arthroplasty	Retrospective, Multi Center	TKA	3	17
2019 June	Milandt et al. [44]	Clinical Orthopaedics and Related Research	A Single Positive Tissue Culture Increases the Risk of Rerevision of Clinically Aseptic THA: A National Register Study	Retrospective, Registry Study	THA	3	21
2020 July	Vargas-Reverón et al. [47]	Journal of Arthroplasty	Prevalence and Impact of Positive Intraoperative Cultures in Partial Hip or Knee Revision	Retrospective, Single Center	THA TKA	3	19
2021 June	Hipfl et al. [35]	The Bone and Joint Journal	Unexpected low-grade infections in revision hip arthroplasty for aseptic loosening: a single-institution experience of 274 hips	Retrospective, Single Center	THA	3	17
2021 August	Neufeld et al. [25]	The Journal of Bone and Joint Surgery	Prevalence and Outcomes of Unexpected Positive Intraoperative Cultures in Presumed Aseptic Revision Hip Arthroplasty	Retrospective, Single Center	THA	3	19
2022 May	Neufeld et al. [48]	Journal of Arthroplasty	The Prevalence and Outcomes of Unexpected Positive Intraoperative Cultures in Presumed Aseptic Revision Knee Arthroplasty	Retrospective, Single Center	TKA	3	20
2022 June	Schwarze et al. [24]	Journal of Arthroplasty	Unsuspected Positive Cultures in Planned Aseptic Revision Knee or Hip Arthroplasty-Risk Factors and Impact on Survivorship	Retrospective, Single Center	THA TKA	3	20

**Table 2:** Follow-up and Intraoperative Sampling.

	Minimum Follow-Up in Months	Follow-Up in Months (Range)	Samples n	Type of Samples
Barrack [33]	24	45 (24–74) ^1^	min. 2	synovial fluid, tissue
Milandt [44]	12	n/a *	3–5	tissue
Vargas-Reverón [47]	60	n/a *	3–6	synovial fluid, tissue
Hipfl [35]	24	68 (26–95) ^1^	min. 3	synovial fluid, tissue, sonication
Neufeld [25]	12	40 (18–77) ^2^	3–5	swabs, synovial fluid, tissue
Neufeld [48]	12	43 (24–74) ^2^	3–5	swabs, synovial fluid, tissue
Schwarze [24]	24	41 (25–31) ^2^	3–5	tissue

^1^ Mean (Range), ^2^ Median (Interquartile Range), * n/a—information not available.

**Table 3:** Preoperative Algorithm for PJI.

Author	Preoperative Algorithm
Barrack [33]	In all cases: Clinical examination, plain radiographs, serum C-reactive protein (CRP), erythrocyte sedimentation rate (ESR) Preoperative synovial fluid aspiration in 1 of 3 centers routinely and in 2 of 3 selectively
Milandt [44]	n/a *
Vargas-Reverón [47]	In all cases: Clinical examination, plain radiographs, serum CRP, ESR, bone scintigraphy If suspicious: synovial fluid aspiration
Hipfl [35]	In all cases: Clinical examination, plain radiographs, serum CRP If suspicious: synovial fluid aspiration
Neufeld [25]	In all cases: Clinical examination, plain radiographs, serum CRP, ESR If suspicious: synovial fluid aspiration
Neufeld [48]	In all cases: Clinical examination, plain radiographs, serum CRP, ESR If suspicious: synovial fluid aspiration
Schwarze [24]	In all cases: Clinical examination, plain radiographs, serum C-reactive protein (CRP), synovial fluid aspiration

* The preoperative workup was not reported in this registry-based study.

**Table 4:** Included cases and UPCs.

	Included Cases n	UPC Total n (% of All Cases)	Single UPC n (% of All UPCs)	Most Frequent Isolated Pathogen (%)
Barrack [33]	692	41 (6)	29 (71)	ConS (49)
Milandt [44]	2305	282 (12)	170 (60)	ConS (67)
Vargas-Reverón [47]	145	48 (32)	35 (73)	ConS (96)
Hipfl [35]	274	77 (28)	41 (53)	ConS (42)
Neufeld [25]	1196	110 (9)	74 (68) *	*C. acnes* (38)
Neufeld [48]	775	76 (10)	62 (82)	*C. acnes* (32)
Schwarze [24]	434	160 (37)	119 (74)	ConS (43)
Total	5821	794 (14)	530 (67)	

* Information on the number of positive cultures was only available in 109/110 cases. *C. acnes*—*Cutibacterium acnes*.

## Data Availability

All underlying data is included in the text, tables, and figures.

## References

[1] Patel A., Pavlou G., Mújica-Mota R.E., Toms A.D. (2015). The epidemiology of revision total knee and hip arthroplasty in England and Wales: A comparative analysis with projections for the United States. A study using the National Joint Registry dataset. Bone Jt. J..

[2] Parvizi J., Tan T.L., Goswami K., Higuera C., Della Valle C., Chen A.F., Shohat N. (2018). The 2018 Definition of Periprosthetic Hip and Knee Infection: An Evidence-Based and Validated Criteria. J. Arthroplast..

[3] Koh C.K., Zeng I., Ravi S., Zhu M., Vince K.G., Young S.W. (2017). Periprosthetic Joint Infection Is the Main Cause of Failure for Modern Knee Arthroplasty: An Analysis of 11,134 Knees. Clin. Orthop. Relat. Res..

[4] Delanois R.E., Mistry J.B., Gwam C.U., Mohamed N.S., Choksi U.S., Mont M.A. (2017). Current Epidemiology of Revision Total Knee Arthroplasty in the United States. J. Arthroplast..

[5] Lum Z.C., Natsuhara K.M., Shelton T.J., Giordani M., Pereira G.C., Meehan J.P. (2018). Mortality During Total Knee Periprosthetic Joint Infection. J. Arthroplast..

[6] Schwarze J., Moellenbeck B., Gosheger G., Schmidt-Braekling T., Lampe L., Klingebiel S., Ackmann T., Theil C. (2021). Poor performance of open incisional biopsy for the microbiological diagnosis of periprosthetic knee joint infection. Sci. Rep..

[7] Kirschbaum S., Erhart S., Perka C., Hube R., Thiele K. (2022). Failure Analysis in Multiple TKA Revisions-Periprosthetic Infections Remain Surgeons’ Nemesis. J. Clin. Med..

[8] Alp E., Cevahir F., Ersoy S., Guney A. (2016). Incidence and economic burden of prosthetic joint infections in a university hospital: A report from a middle-income country. J. Infect. Public Health.

[9] Weber M., Renkawitz T., Voellner F., Craiovan B., Greimel F., Worlicek M., Grifka J., Benditz A. (2018). Revision Surgery in Total Joint Replacement Is Cost-Intensive. BioMed Res. Int..

[10] Sabah S.A., Knight R., Alvand A., Murray D.W., Petrou S., Beard D.J., Price A.J. (2022). No exponential rise in revision knee replacement surgery over the past 15 years: An analysis from the National Joint Registry. Osteoarthr. Cartil..

[11] Parvizi J., Zmistowski B., Berbari E.F., Bauer T.W., Springer B.D., Della Valle C.J., Garvin K.L., Mont M.A., Wongworawat M.D., Zalavras C.G. (2011). New definition for periprosthetic joint infection: From the Workgroup of the Musculoskeletal Infection Society. Clin. Orthop. Relat. Res..

[12] Osmon D.R., Berbari E.F., Berendt A.R., Lew D., Zimmerli W., Steckelberg J.M., Rao N., Hanssen A., Wilson W.R. (2013). Executive summary: Diagnosis and management of prosthetic joint infection: Clinical practice guidelines by the Infectious Diseases Society of America. Clin. Infect. Dis..

[13] McNally M., Sousa R., Wouthuyzen-Bakker M., Chen A.F., Soriano A., Vogely H.C., Clauss M., Higuera C.A., Trebše R. (2021). The EBJIS definition of periprosthetic joint infection. Bone Jt. J..

[14] Tubb C.C., Polkowksi G.G., Krause B. (2020). Diagnosis and Prevention of Periprosthetic Joint Infections. J. Am. Acad. Orthop. Surg..

[15] Parvizi J., Gehrke T., Mont M.A., Callaghan J.J. (2019). Introduction: Proceedings of International Consensus on Orthopedic Infections. J. Arthroplast..

[16] Sigmund I.K., Luger M., Windhager R., McNally M.A. (2022). Diagnosing periprosthetic joint infections: A comparison of infection definitions: EBJIS 2021, ICM 2018, and IDSA 2013. Bone Jt. Res..

[17] Romanò C.L., Khawashki H.A., Benzakour T., Bozhkova S., Del Sel H., Hafez M., Johari A., Lob G., Sharma H.K., Tsuchiya H. (2019). The W.A.I.O.T. Definition of High-Grade and Low-Grade Peri-Prosthetic Joint Infection. J. Clin. Med..

[18] Vasso M., Schiavone Panni A. (2015). Low-grade periprosthetic knee infection: Diagnosis and management. J. Orthop. Traumatol..

[19] Balato G., Franceschini V., Ascione T., Lamberti A., Balboni F., Baldini A. (2018). Diagnostic accuracy of synovial fluid, blood markers, and microbiological testing in chronic knee prosthetic infections. Arch. Orthop. Trauma Surg..

[20] Partridge D.G., Winnard C., Townsend R., Cooper R., Stockley I. (2018). Joint aspiration, including culture of reaspirated saline after a ‘dry tap’, is sensitive and specific for the diagnosis of hip and knee prosthetic joint infection. Bone Jt. J..

[21] Enz A., Becker J., Warnke P., Prall F., Lutter C., Mittelmeier W., Klinder A. (2020). Increased Diagnostic Certainty of Periprosthetic Joint Infections by Combining Microbiological Results with Histopathological Samples Gained via a Minimally Invasive Punching Technique. J. Clin. Med..

[22] Peel T.N., Spelman T., Dylla B.L., Hughes J.G., Greenwood-Quaintance K.E., Cheng A.C., Mandrekar J.N., Patel R. (2017). Optimal Periprosthetic Tissue Specimen Number for Diagnosis of Prosthetic Joint Infection. J. Clin. Microbiol..

[23] Bémer P., Léger J., Tandé D., Plouzeau C., Valentin A.S., Jolivet-Gougeon A., Lemarié C., Kempf M., Héry-Arnaud G., Bret L. (2016). How Many Samples and How Many Culture Media To Diagnose a Prosthetic Joint Infection: A Clinical and Microbiological Prospective Multicenter Study. J. Clin. Microbiol..

[24] Schwarze J., Dieckmann R., Gosheger G., Bensmann M., Moellenbeck B., Theil C. (2022). Unsuspected Positive Cultures in Planned Aseptic Revision Knee or Hip Arthroplasty-Risk Factors and Impact on Survivorship. J. Arthroplast..

[25] Neufeld M.E., Lanting B.A., Shehata M., Howard J.L., MacDonald S.J., Teeter M.G., Vasarhelyi E.M. (2021). Prevalence and Outcomes of Unexpected Positive Intraoperative Cultures in Presumed Aseptic Revision Hip Arthroplasty. J. Bone Jt. Surg. Am..

[26] Parvizi J., Suh D.-H., Jafari S.M., Mullan A., Purtill J.J. (2011). Aseptic loosening of total hip arthroplasty: Infection always should be ruled out. Clin. Orthop. Relat. Res..

[27] Pedersen A.B., Svendsson J.E., Johnsen S.P., Riis A., Overgaard S. (2010). Risk factors for revision due to infection after primary total hip arthroplasty. A population-based study of 80,756 primary procedures in the Danish Hip Arthroplasty Registry. Acta Orthop..

[28] Yazdi H., Restrepo C., Foltz C., Hammad M., Chung P.H., Gomella L.G., Parvizi J. (2020). Symptomatic Benign Prostatic Hyperplasia: A Risk Factor for Periprosthetic Joint Infection in Male Patients. J. Bone Jt. Surg. Am..

[29] Panula V.J., Alakylä K.J., Venäläinen M.S., Haapakoski J.J., Eskelinen A.P., Manninen M.J., Kettunen J.S., Puhto A.-P., Vasara A.I., Elo L.L. (2021). Risk factors for prosthetic joint infections following total hip arthroplasty based on 33,337 hips in the Finnish Arthroplasty Register from 2014 to 2018. Acta Orthop..

[30] Kunutsor S.K., Whitehouse M.R., Blom A.W., Beswick A.D. (2016). Patient-Related Risk Factors for Periprosthetic Joint Infection after Total Joint Arthroplasty: A Systematic Review and Meta-Analysis. PLoS ONE.

[31] Staats K., Kolbitsch P., Sigmund I.K., Hobusch G.M., Holinka J., Windhager R. (2017). Outcome of Total Hip and Total Knee Revision Arthroplasty With Minor Infection Criteria: A Retrospective Matched-Pair Analysis. J. Arthroplast..

[32] Gallo J., Nieslanikova E. (2020). Prevention of Prosthetic Joint Infection: From Traditional Approaches towards Quality Improvement and Data Mining. J. Clin. Med..

[33] Barrack R.L., Aggarwal A., Burnett R.S.J., Clohisy J.C., Ghanem E., Sharkey P., Parvizi J. (2007). The fate of the unexpected positive intraoperative cultures after revision total knee arthroplasty. J. Arthroplast..

[34] Saleh A., Guirguis A., Klika A.K., Johnson L., Higuera C.A., Barsoum W.K. (2014). Unexpected positive intraoperative cultures in aseptic revision arthroplasty. J. Arthroplast..

[35] Hipfl C., Mooij W., Perka C., Hardt S., Wassilew G.I. (2021). Unexpected low-grade infections in revision hip arthroplasty for aseptic loosening: A single-institution experience of 274 hips. Bone Jt. J..

[36] Jacobs A.M.E., Bénard M., Meis J.F., van Hellemondt G., Goosen J.H.M. (2017). The unsuspected prosthetic joint infection: Incidence and consequences of positive intra-operative cultures in presumed aseptic knee and hip revisions. Bone Jt. J..

[37] Boot W., Moojen D.J.F., Visser E., Lehr A.M., de Windt T.S., van Hellemondt G., Geurts J., Tulp N.J.A., Schreurs B.W., Burger B.J. (2015). Missed low-grade infection in suspected aseptic loosening has no consequences for the survival of total hip arthroplasty. Acta Orthop..

[38] Fernandez-Sampedro M., Salas-Venero C., Fariñas-Álvarez C., Sumillera M., Pérez-Carro L., Fakkas-Fernandez M., Gómez-Román J., Martínez-Martínez L., Fariñas M.C. (2015). 26Postoperative diagnosis and outcome in patients with revision arthroplasty for aseptic loosening. BMC Infect. Dis..

[39] Ribera A., Morata L., Moranas J., Agulló J.L., Martínez J.C., López Y., García D., Cabo J., García-Ramiro S., Soriano A. (2014). Clinical and microbiological findings in prosthetic joint replacement due to aseptic loosening. J. Infect..

[40] Marculescu C.E., Berbari E.F., Hanssen A.D., Steckelberg J.M., Osmon D.R. (2005). Prosthetic joint infection diagnosed postoperatively by intraoperative culture. Clin. Orthop. Relat. Res..

[41] Gundtoft P.H., Pedersen A.B., Schønheyder H.C., Overgaard S. (2016). Validation of the diagnosis ‘prosthetic joint infection’ in the Danish Hip Arthroplasty Register. Bone Jt. J..

[42] Schwarze J., Theil C., Gosheger G., Dieckmann R., Moellenbeck B., Ackmann T., Schmidt-Braekling T. (2020). Promising results of revision total hip arthroplasty using a hexagonal, modular, tapered stem in cases of aseptic loosening. PLoS ONE.

[43] Theil C., Freudenberg S.C., Gosheger G., Schmidt-Braekling T., Schwarze J., Moellenbeck B. (2020). Do Positive Cultures at Second Stage Re-Implantation Increase the Risk for Reinfection in Two-Stage Exchange for Periprosthetic Joint Infection?. J. Arthroplast..

[44] Milandt N.R., Gundtoft P.H., Overgaard S. (2019). A Single Positive Tissue Culture Increases the Risk of Rerevision of Clinically Aseptic THA: A National Register Study. Clin. Orthop. Relat. Res..

[45] Page M.J., McKenzie J.E., Bossuyt P.M., Boutron I., Hoffmann T.C., Mulrow C.D., Shamseer L., Tetzlaff J.M., Akl E.A., Brennan S.E. (2021). The PRISMA 2020 statement: An updated guideline for reporting systematic reviews. BMJ.

[46] Slim K., Nini E., Forestier D., Kwiatkowski F., Panis Y., Chipponi J. (2003). Methodological index for non-randomized studies (minors): Development and validation of a new instrument. ANZ J. Surg..

[47] Vargas-Reverón C., Soriano A., Fernández-Valencia J.A., Martínez-Pastor J.C., Morata L., Muñoz-Mahamud E. (2020). Prevalence and Impact of Positive Intraoperative Cultures in Partial Hip or Knee Revision. J. Arthroplast..

[48] Neufeld M.E., Lanting B.A., Shehata M., Naudie D.D.R., McCalden R.W., Teeter M.G., Vasarhelyi E.M. (2022). The Prevalence and Outcomes of Unexpected Positive Intraoperative Cultures in Presumed Aseptic Revision Knee Arthroplasty. J. Arthroplast..

[49] Theil C., Schneider K.N., Gosheger G., Schmidt-Braekling T., Ackmann T., Dieckmann R., Frommer A., Klingebiel S., Schwarze J., Moellenbeck B. (2022). Revision TKA with a distal femoral replacement is at high risk of reinfection after two-stage exchange for periprosthetic knee joint infection. Knee Surg. Sport. Traumatol. Arthrosc..

[50] Rothenberg A.C., Wilson A.E., Hayes J.P., O’Malley M.J., Klatt B.A. (2017). Sonication of Arthroplasty Implants Improves Accuracy of Periprosthetic Joint Infection Cultures. Clin. Orthop. Relat. Res..

[51] Trampuz A., Piper K.E., Jacobson M.J., Hanssen A.D., Unni K.K., Osmon D.R., Mandrekar J.N., Cockerill F.R., Steckelberg J.M., Greenleaf J.F. (2007). Sonication of removed hip and knee prostheses for diagnosis of infection. N. Engl. J. Med..

[52] Krenn V., Morawietz L., Perino G., Kienapfel H., Ascherl R., Hassenpflug G.J., Thomsen M., Thomas P., Huber M., Kendoff D. (2014). Revised histopathological consensus classification of joint implant related pathology. Pathol. Res. Pract..

